# Climate Change is Likely to Worsen the Public Health Threat of Diarrheal Disease in Botswana

**DOI:** 10.3390/ijerph10041202

**Published:** 2013-03-26

**Authors:** Kathleen A. Alexander, Marcos Carzolio, Douglas Goodin, Eric Vance

**Affiliations:** 1 Fisheries and Wildlife Conservation, Virginia Tech, 100 Cheatham Hall, Blacksburg, VA 24061, USA; 2 CARACAL, Plot 110, Kasane, Botswana; 3 Department of Statistics, Virginia Tech, 405C Hutcheson Hall, Blacksburg, VA 24061, USA; E-Mails: cmarcos8@vt.edu (M.C.); ervance@vt.edu (E.V.); 4 Department of Geography, Kansas State University, 122 Seaton Hall, Manhattan, KS 66506, USA; E-Mail: dgoodin@ksu.edu

**Keywords:** climate change, infectious disease, diarrhea, water, sanitation, hygiene, Africa, Botswana, pathogen transmission, passive surveillance

## Abstract

Diarrheal disease is an important health challenge, accounting for the majority of childhood deaths globally. Climate change is expected to increase the global burden of diarrheal disease but little is known regarding climate drivers, particularly in Africa. Using health data from Botswana spanning a 30-year period (1974–2003), we evaluated monthly reports of diarrheal disease among patients presenting to Botswana health facilities and compared this to climatic variables. Diarrheal case incidence presents with a bimodal cyclical pattern with peaks in March (ANOVA *p* < 0.001) and October (ANOVA *p* < 0.001) in the wet and dry season, respectively. There is a strong positive autocorrelation (*p* < 0.001) in the number of reported diarrhea cases at the one-month lag level. Climatic variables (rainfall, minimum temperature, and vapor pressure) predicted seasonal diarrheal with a one-month lag in variables (*p* < 0.001). Diarrheal case incidence was highest in the dry season after accounting for other variables, exhibiting on average a 20% increase over the yearly mean (*p* < 0.001). Our analysis suggests that forecasted climate change increases in temperature and decreases in precipitation may increase dry season diarrheal disease incidence with hot, dry conditions starting earlier and lasting longer. Diarrheal disease incidence in the wet season is likely to decline. Our results identify significant health-climate interactions, highlighting the need for an escalated public health focus on controlling diarrheal disease in Botswana. Study findings have application to other arid countries in Africa where diarrheal disease is a persistent public health problem.

## 1. Introduction

Climate change is predicted to have a strong influence on human health with the most vulnerable communities being those living in poverty and having the lowest capacity to adapt [1]. Sub-Saharan Africa is a particularly vulnerable region as it has the highest burden of infectious disease [2] and is projected to be the most affected by climate change [1]. Understanding climate variability as a determinant of infectious disease is increasingly seen as a cornerstone of climate change preparedness and an urgent area of need in Africa. 

Diarrheal disease represents an important disease syndrome that is predicted to be particularly impacted by climate change [3]. Globally, diarrheal illness remains one of the leading causes of morbidity and mortality, with the majority of deaths occurring in children under 5 years of age [4]. It is estimated that diarrheal disease accounts for 15% of all childhood deaths for this age group, the majority occurring in the developing world, particularly in Africa [5]. Previous studies have found an array of climatic factors associated with diarrheal disease including temperature, rainfall, relative humidity, and air pressure (reviewed by [6]), which may vary by region (e.g., [7,8,9]). Lack of empirical data has led, however, to great uncertainty regarding the nature of potential climate impacts [6], a gap requiring urgent attention. This is particularly important for vulnerable regions where low adaptive capacity might exist due to underlying conditions of poor governance, poverty, and weak resource management. 

Historical data provides a rich opportunity to evaluate climate-health interactions. However, long-term time series data are often not available and confounders over a similar study period are infrequently identified at the same spatial and temporal scale [9]. This is particularly true in Africa, where long-term health data are often lost or inconsistently collected due to weak health infrastructure and poor data recording and archiving. The few empirical studies that have been undertaken in Africa have relied on limited time series data to derive climate-diarrhea incidence relationships [10,11]. Extreme variation in climate over time [12] and the complexity of diarrheal disease causation complicates the use of such limited data sets to derive climate-health relationships for a particular region. With few exceptions, longer term data sets are identified from high income countries [13], focusing research in areas of the world with greater economic potential to mitigate and adapt to the effects of climate change both socially and environmentally. Understanding the potential health impacts of climate change in low-income countries will be essential to developing mitigation and adaptive strategies designed to protect these vulnerable populations expected to be impacted the hardest and likely the least able to adapt [14,15].

Our study focuses on Botswana, an arid country located in Southern Africa. Climate change impacts on diarrheal disease and population vulnerability are expected to be higher among human populations living in such water-restricted environments [15]. In Botswana, diarrheal disease is reported to be the second most important cause of mortality in children under the age of 5 [5]. Using a unique thirty-year data set (1974–2003), we provide the first reported multi-decadal analysis of diarrheal disease and its associations with meteorological drivers and climate in sub-Saharan Africa. We discuss our findings in light of climate change projections and assess implications to public health preparedness in Botswana and other arid countries similarly affected by diarrheal disease. 

## 2. Experimental Section

### 2.1. Study site and Associated Data Sets

**Figure 1:**
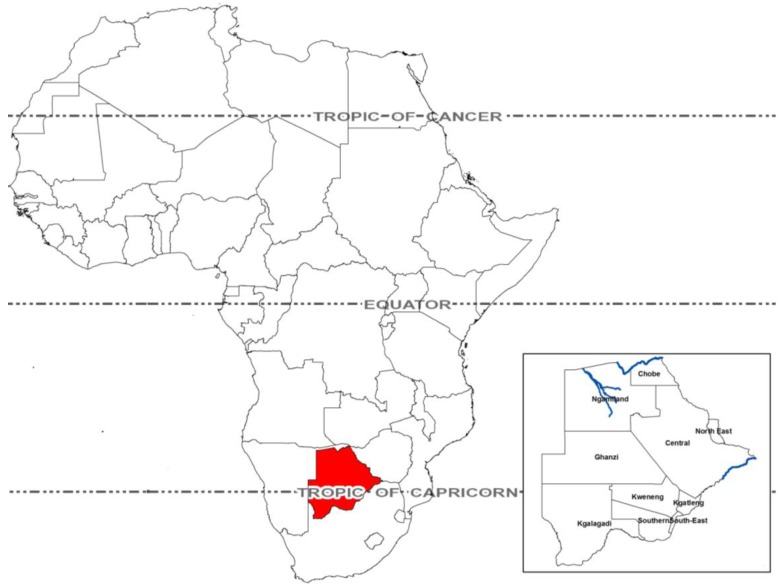
This study was conducted in Botswana (red) a landlocked country located largely within the tropics. This arid country has limited surface water with only three permanent sources (inset, blue) occurring in the country.

Botswana is a politically stable, semi-arid, landlocked country located in sub-Saharan Africa (Figure 1). The country has a subtropical climate with annual wet (November–March) and dry (April–October) seasons. Only three permanent sources of surface water occur, all originating outside the country (94% of all river flow), increasing vulnerability to regional water abstraction, decreased flow, and water scarcity [16]. Rainfall can be extremely limited and is highly variable in the country both within and between years, with recurrent occurrence of both flooding and droughts [17], the latter appearing to occur on a 10 to12 years cycle [18]. 

Piped water is available for most of the population either through direct reticulation or provision of water through public taps. At the time of independence in 1966, Botswana was one of the poorest countries in the world. With the discovery of mineral wealth, Botswana has seen rapid economic growth and is now considered an upper middle-income economy [19]. Large investments in health care have occurred with services freely available to the public with a nominal charge (<1 United States dollar) only recently applied. 

#### 2.1.1. Temperature and Vapor Pressure

Time series data of minimum temperature (T_min_), maximum temperature (T_max_), average temperature (T_mean_), diurnal temperature range (DTR), rainfall (for secondary evaluation against the derived interpolated data set see below), and vapor pressure (q) for the period 1974–2003 were extracted from the Climate Research Unit (CRU) TS 3.10 data set, archived at the Royal Netherlands Meteorological Institute’s (KNMI) Climate Explorer site (http://climexp.knmi.nl/). Data from the CRU TS 3.10 set are stored as monthly gridded averages for 0.5° × 0.5° cells (see [20] for a comprehensive discussion of the methods used to create these products). We created our time series by identifying every grid cell located completely within Botswana’s borders, then averaging individual cell values to create a single value of each variable for the entire country. Climate data were summarized in this manner to compare to the health dataset, which was only available at a national level by month. 

In addition to the time series values listed above, we also created a derived climate index, T_min_/q. T_min_/q represents the ratio of each month’s minimum temperature with the average vapor pressure (q) for that month. T_min_/q functions as a rough climatological index for aridity, which is of particular importance in this analysis, given the potential relationship between aridity and diarrheal disease risk [15]. Minimum temperature is used in this index because in systems where the daily temperature wave is controlled mainly by radiative and thermal drivers (rather than by advective effects) it should correspond roughly to the dew point temperature. Normalizing T_min_ via the vapor pressure (a measure of the specific humidity), therefore, provides an index of dryness that is less dependent on the effects of temperature [21,22].

#### 2.1.2. Gridded Rainfall Data Set

Monthly rainfall data were collected from 1974–2008 from 53 rain gauges located across Botswana (Meteorology Department, Botswana Government). The Inverse Distance Weighted (IDW) in ArcGIS 10 (Environmental Systems Research Institute (ERSI), Redlands, CA, USA) method was used to interpolate monthly rainfall data for the entire country. IDW assumes that each sample point has a local influence and decreases with distance and, thus, weighs points closer to the processing cell more than points farther away. This method is appropriate when the variable being mapped decreases in influence as distance from the sampled location increases and topographic/orographic influences are minimal. IDW is an exact deterministic interpolator. Optimal parameters were chosen by individually analyzing each month. ArcGIS 10 Geostatistical Wizard was used to estimate the optimal power for each layer by finding the number that minimizes the root mean square error value. The powers that were used ranged between 1 and 2.46, with a mean of 1.44. The minimum neighboring points used was 5 and the maximum was 6 for all analyses. Precipitation data extracted from CRU were very similar to the interpolated rainfall data generated in this present study (Pearson coefficient r = 0.92, p < 0.001). Rainfall data derived from the Botswana Interpolation were then used in all further analyses. 

#### 2.1.3. Diarrheal Case Incidence and Medical Facilities (1974–2003)

We extracted the number of diarrheal patients presenting to government health facilities in Botswana from hard copy annual health reports prepared by the Central Statistics Office. These case data are compiled by the Ministry of Health (1974–2003) under the Botswana Government. Case data represent summary clinical diagnoses of attending physicians or nurses in Government medical facilities. Diagnoses were categorized from 1979 onwards according to the International Classification of Disease (starting with ICD 7 [23]) and modified according to the most recently published ICD revision. Previous to this date, the cases were based on Government identified disease categories (1974–1978). Reported case data were not associated with any clinical diagnostic information but rather represent summary diagnoses categorized as specified above. 

Data were unavailable for the years 1976, 1979, 1981, and 1983. These reports were located in various government offices and libraries to reconstruct a long-term health data set. Data were manually extracted from hard copy reports and digitized. Located reports started in 1974 when the Government of Botswana established an annual health reporting system, eight years after independence from British rule. 

Annual reports reflect the summary diagnoses of patients attending government hospitals and clinics across the nation over the year and are reported at the aggregated national level on a monthly basis. Diarrheal case incidence is then calculated as the number of cases of diarrhea reported by month per thousand population for the respective year [24]. Population data (1974–2003) were extracted from the United States Census Bureau’s International Data Base [25]. Data were crosschecked against limited Botswana census data collected by the Central Statistics Office (www.cso.gov). 

The numbers of health facilities established over time were derived from these same health reports and represent the combined number of hospitals and clinics open to the public by year from 1974 to 2003. Mobile clinic stops in remote areas were not included due to the great variation in reported values and potential bias in data reporting. To evaluate the potential bias arising from health facility development on diarrhea case incidence, a simple linear regression was performed between the two variables. No human subjects work was undertaken in this study as these government reports were prepared public reports. All data were anonymized.

### 2.2. Statistical Analysis

All statistical analyses in this study were conducted using R [26] and the “MASS” library [27]. Model selection was done using a forward-backward stepwise variable selection approach to minimize the Bayesian information criterion [28]. This method penalizes models with too many variables while maximizing the model likelihood, thereby attempting to avoid an over-fitting of the data.

#### 2.2.1. Monthly Analysis

We analyzed monthly diarrhea against predictor variables using an autoregressive analysis of covariance model (ANCOVA [29]). Autoregressive analyses are typically used to model time series data that depend linearly on their previous time step. Diarrheal patterns are suited for this method precisely because we suspect a time dependence structure for disease outbreaks might be present. Additionally, analysis of covariance is appropriate since all model variables are either categorical or continuous covariates.

The analysis included monthly average national rainfall, vapor pressure, inverse vapor pressure (1/q), a dummy variable indicating wet or dry season, temperature variables, and minimum temperature divided by vapor pressure (T_min_/q). All variables were lagged by one month except for the season indicator, meaning that we used the previous month’s variables to predict the subsequent month’s diarrhea. The one-month lag in climate variables is a direct consequence of the stepwise variable selection procedure, a result we attribute to the possibly delayed response time of disease dynamics in relation to weather events.

Dealing with socioeconomic variation over time can add an important amount of complexity to the analysis of long-term, time series data. To minimize potential reporting biases associated with these changes and other potential confounding variables, we performed our analyses by standardizing monthly diarrheal data on a yearly basis, transforming the data as follows:

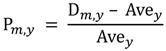
(1)
where P_m,y_ is defined as the monthly proportion deviation from the mean number of cases of diarrhea for month *m* in year *y*, D_m,y_ is the number of reported cases of diarrhea in month *m* of year *y*, and Ave_y_ is the mean number of monthly cases of diarrhea for year *y*. In other words, we take each month of a given year and subtract the mean diarrhea case incidence of that particular year from the diarrheal case incidence for that month, giving us the proportion deviation of diarrhea case incidence for a particular month. This standardized value is used to compare diarrhea case incidence over time, minimizing the confounding influence of socioeconomic development and other variables that might have a longitudinal impact on diarrheal incidence. 

#### 2.2.2. Missing Data Considerations

Four years of national diarrhea data were unavailable for our analyses: 1976, 1979, 1981, and 1983. As a result, we impute an estimate of the proportion deviation from the yearly mean diarrhea for December of those missing years, allowing us to use these values in the prediction of diarrhea in January of the following year. Our final sample size, including the four imputed data points, was 315 months.

## 3. Results

### 3.1. Rainfall

**Figure 2:**
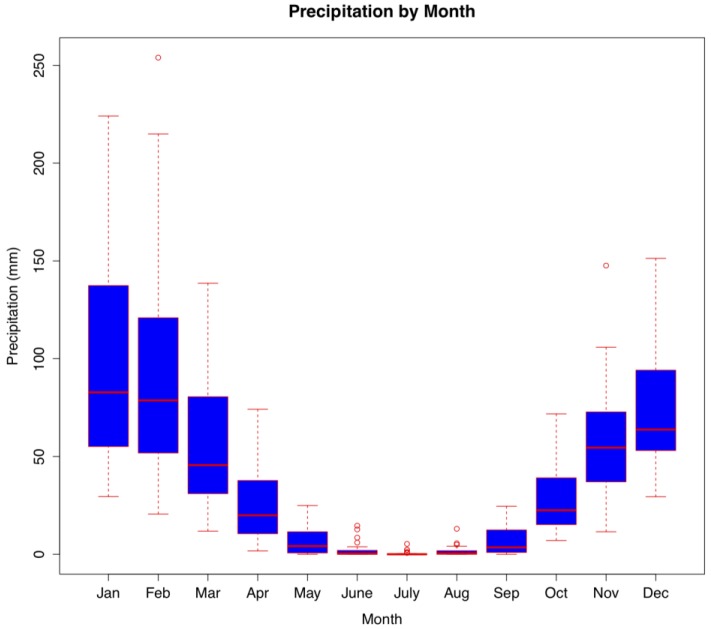
Botswana has a subtropical climate with clear wet and dry seasons. The wet season is from November to March, as is evident from these box-and-whisker plots of monthly rainfall from 1974 to 2003.

**Figure 3:**
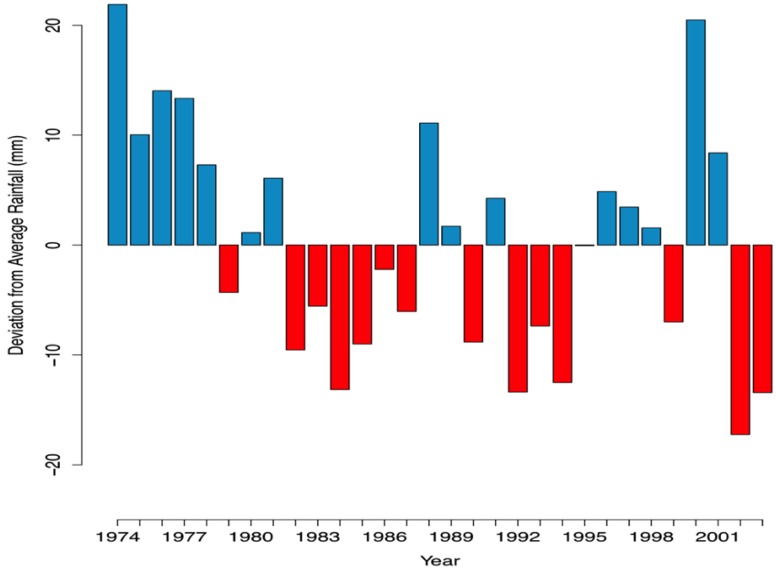
Annual rainfall patterns in Botswana from 1974–2003 reflect high variability between years and the occurrence of drought (red) and wet cycles (blue).

Monthly rainfall in Botswana (1974–2003) ranged from 0 to 254 mm, with an annual average of 36.97 ± 44.15 mm, and with clear wet and dry seasonal patterns associated with the subtropical climate of Botswana (Figure 2). Over 46% of the years in our dataset showed at least one month with no rainfall and at least one other month with more than 100 mm of precipitation. Annual and seasonal rainfall can vary substantially with periodic dry and wet periods being identified over time within and between years (Figure 2, Figure 3). 

### 3.2. Health Facilities and Medical Human Resources Development

**Figure 4:**
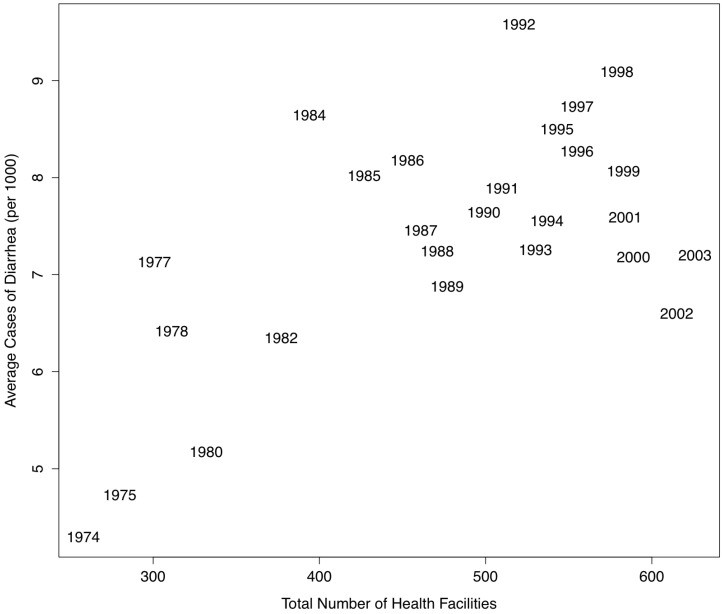
Total number of health facilities has increased in Botswana from 1974–2003 as has reporting of diarrheal disease during this period, identifying an important potential bias in longitudinal data derived from disease case reports.

**Figure 5:**
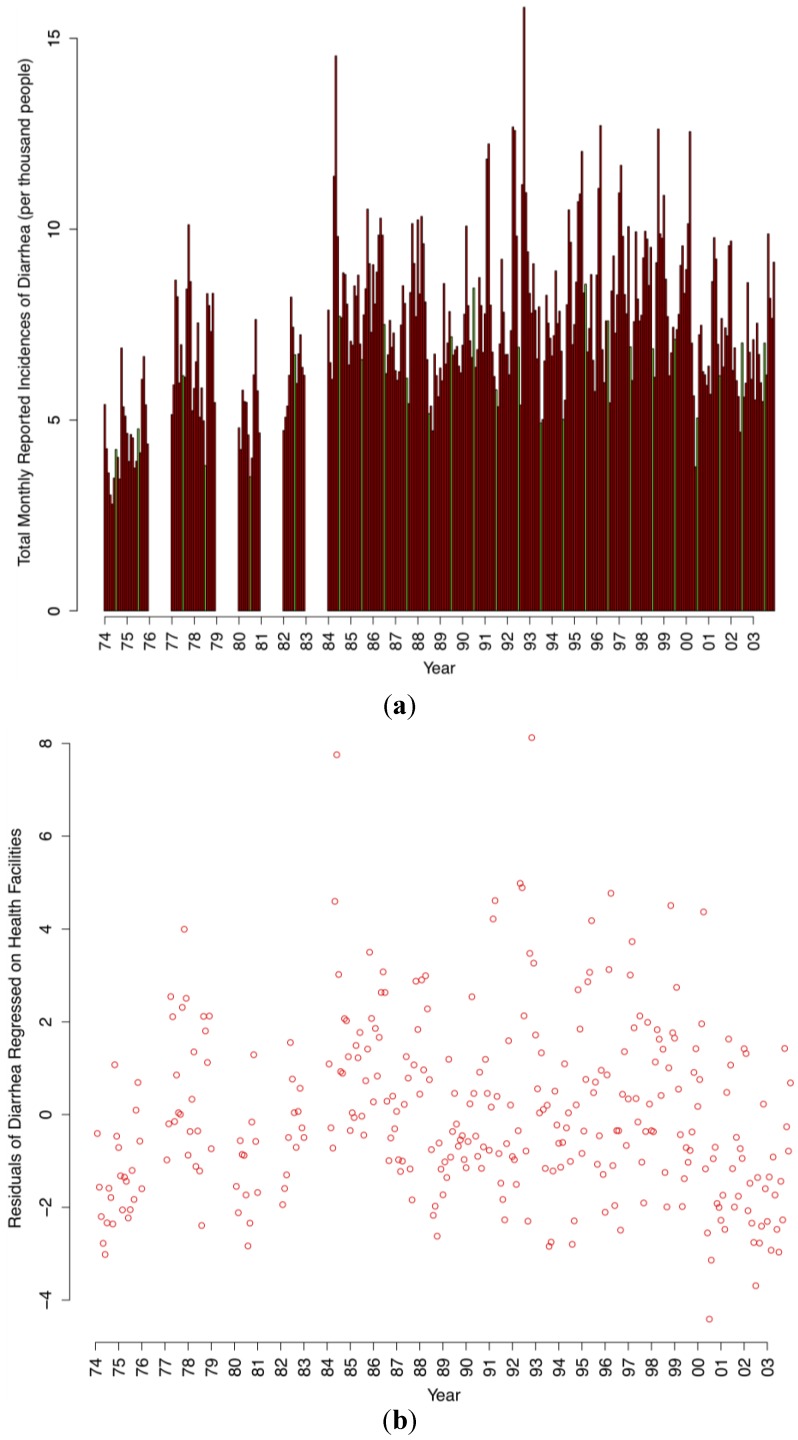
(**a**) Diarrheal case incidence among health facilities in Botswana from 1974–2003. Green bars indicate July, which occurs in the cold dry season. (**b**). Residuals of diarrheal case incidence are regressed against the number of health facilities developed over time providing a measure of variability in case incidence unaccounted for by growth in the number of health facilities.

Hospital and clinic facilities have increased significantly over time in Botswana from 13 hospitals and 47 clinics in 1974 to 33 hospitals and 257 clinics in 2003. There is a positive relationship between total health facility development and diarrheal cases by year (*p* = 0.002, Figure 4, Figure 5).

### 3.3. Diarrhea in Botswana

#### 3.3.1. Long-Term Annual Trends in Botswana (1974–2003)

Over the 30 years period (n = 26 years of data), annual diarrhea incidence averaged 88.48 ± 16 cases per year per 1,000 people (Figure 5 (a)). The highest number occurred in 1992 (115 cases per thousand, corrected for increases in health facilities). 

#### 3.3.2. Trends in Monthly Diarrheal Disease

**Figure 6:**
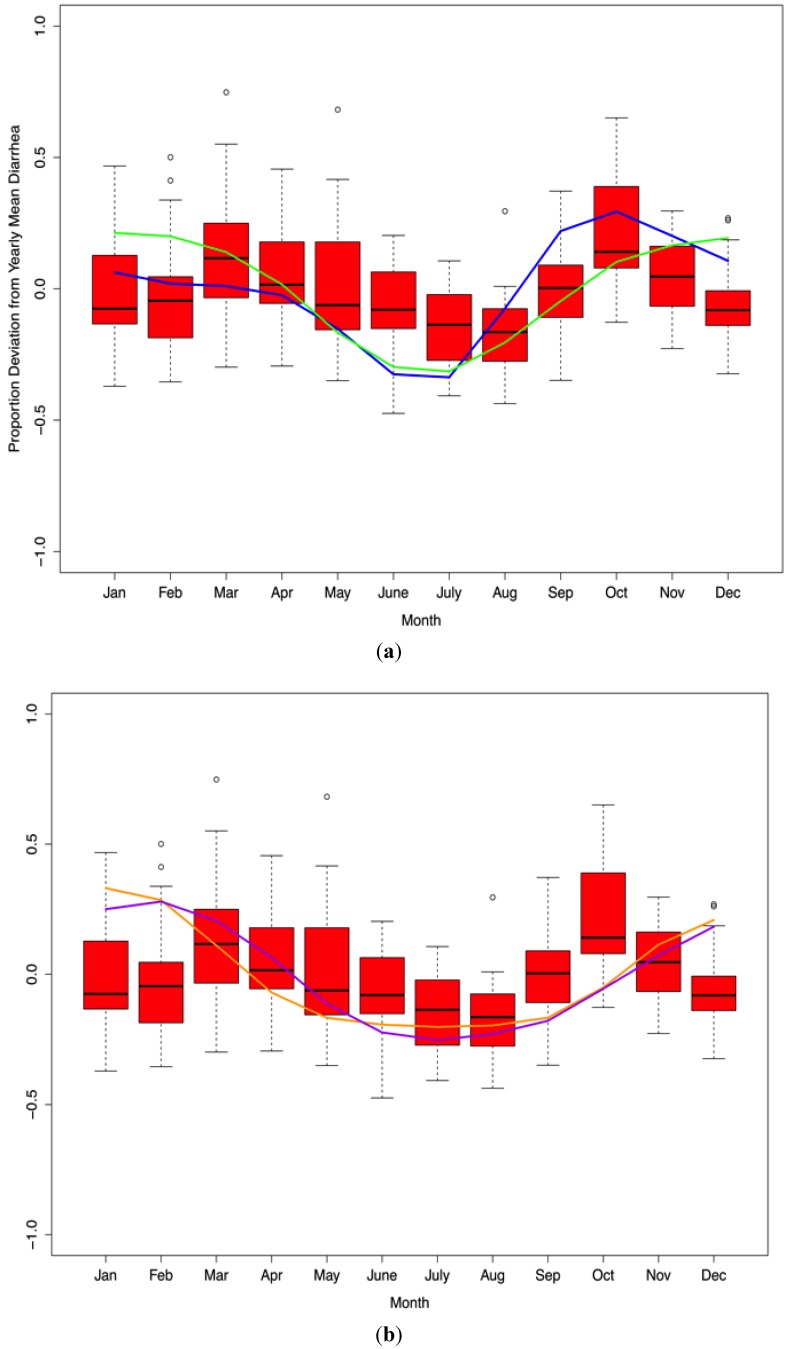
(**a**) Standardized minimum temperature (green line) and minimum temperature/vapor pressure (blue line) are plotted over diarrhea case incidence presented as a proportion deviation from yearly mean diarrhea cases (red box plot). (**b**) Standardized rainfall (orange line) and vapor pressure (purple line) are plotted in a similar manner with diarrhea cases. Together, these meteorological variables act as strong predictors of average monthly diarrhea in Botswana (1974–2003).

At the monthly level, diarrheal case incidence presents with a bimodal cyclical pattern, with significant peaks in March (ANOVA *p* < 0.001) and October (ANOVA *p* < 0.001, Figure 6). However, October represented the peak month of diarrheal case incidence on average over the study period. There is a strong positive autocorrelation (*p* < 0.001) in reported diarrheal case incidences at the one-month lag level (lag1). When accounting for the previous month’s diarrhea cases, rainfall, minimum temperature, and vapor pressure quantities, there was, on average, a 20% increase over the yearly average of diarrheal cases reported in the dry season than the wet season (*p* < 0.001).

### 3.4. Climatic Drivers of Diarrhea

#### 3.4.1. Rainfall and Diarrheal Disease

When total annual diarrheal case incidence is compared to total annual rainfall (inclusive of reporting increases associated with health facility development), we find no significant rainfall effect (*p* = 0.7). However, in our full model, which accounts for the wet and dry seasons, commensurate with a subtropical type climate found in Botswana, we find that rainfall in the prior month has a significant, slightly positive effect on the subsequent month’s diarrheal case incidence in the wet season (*p* = 0.003). In this, the seasonal effect of rainfall is identified. 

#### 3.4.2. Temperature (Minimum, Maximum, Diurnal Temperature Range), Rainfall, Vapor Pressure, and Diarrheal Disease and Season (Wet and Dry)

When using the Bayesian information criterion and accounting for a one-month lag in diarrhea, the first-order autoregressive ANCOVA model that best fit to monthly diarrheal case incidence over the study period included a dummy variable for season and the following lagged climate variables: minimum temperature (T_min_), vapor pressure (q), the derived variable T_min_/q, and rainfall (*p* < 0.001, Figure 6). Other temperature variables were not significant (average temperature, maximum temperature, and DTR). As rainfall and/or Tmin/q increases so do the expected number of cases of diarrhea (*p* < 0.001). Vapor pressure has an influence on both wet and dry season diarrhea (*p* < 0.001) in a bidirectional manner with high vapor pressure increasing diarrheal disease in the wet season and decreasing diarrheal disease in the dry season (Figure 6). Using this procedure, the best model for monthly diarrheal case incidence is mathematically described in Equation (2):

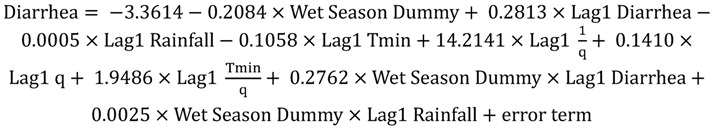
(2)


Here, “Wet Season Dummy” is a dummy variable, taking the value of 1 in the wet season and 0 in the dry season. “T_min_” is the minimum temperature, and “q” is the vapor pressure. “Lag1” as a prefix indicates that the variable used was the previous month’s number.

**Figure 7:**
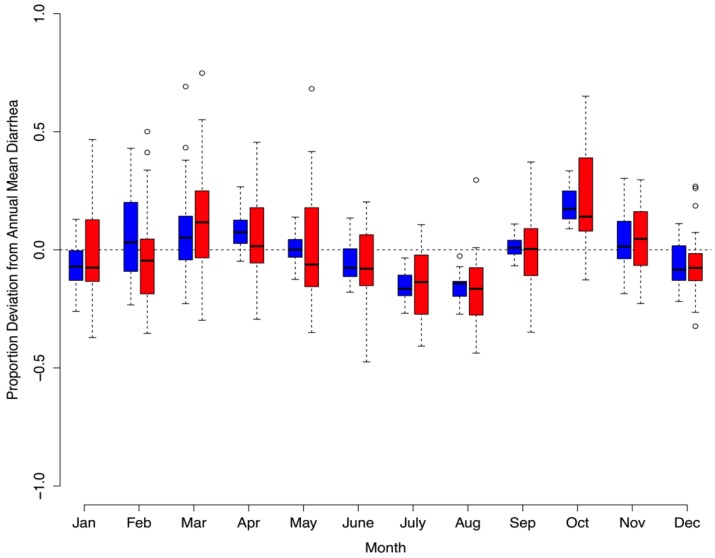
Comparison between predicted monthly values (blue box plots) and observed monthly values (red box plots) for the proportion deviation from the yearly average diarrhea in Botswana from 1974–2003.

**Figure 8:**
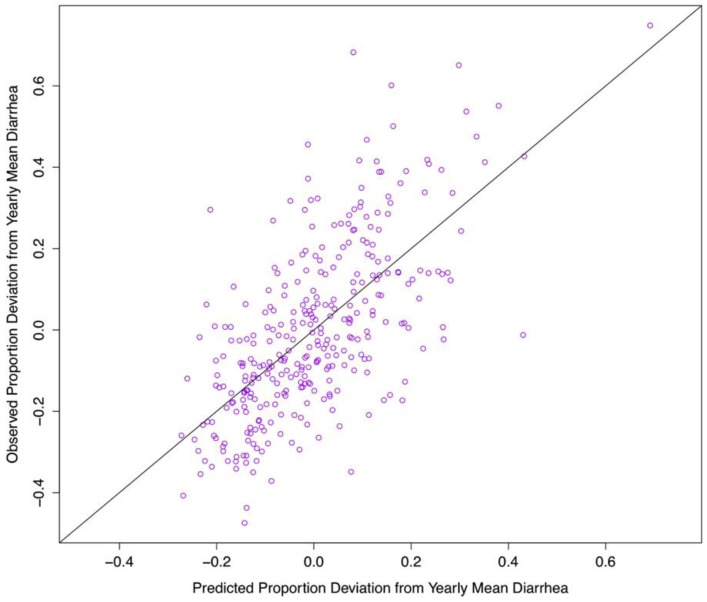
Predicted proportion deviation from yearly average diarrhea in Botswana (1974–2003) plotted against the actual data. Holding out the second half of the dataset and using the first half to estimate the model and predict the second set yields a predicted sum of squares statistic of 0.58. A perfect prediction would line up exactly on the 45° line.

When evaluating model performance, we find that the model captures the bimodal nature of seasonal diarrheal patterns in Botswana (Figure 7). By holding out the second half of our dataset, we can use the first half of the data (1974–1988) to estimate model coefficients, then use the model to predict diarrhea in the second half of the dataset (1989–2003), as depicted in Figure 8. This holdout process yields a predicted residual sum of squares (PRESS) = 0.58, suggesting that our model can accurately predict up to 58% of the variability of future diarrhea. 

To provide some insight into the interpretation of climate variable effects by season, we consider the average scenario by incrementally changing one climate variable in the model to evaluate impacts on diarrheal case incidence (presented as a proportion deviation from yearly mean, Pm,y) holding all other climate variables constant (Table 1). 

**Table 1:** Modeled predictions of climate variable influence on diarrheal disease incidence (presented as a proportion deviation from yearly mean, Pm,y) are illustrated by increasing each variable respectively by one half of a standard deviation (SD) from the mean seasonal value while holding all other variables constant.

Climate Variable	Incremental Effect in Wet Season Pm, y	Incremental Effect in Dry Season Pm, y
Increased by 0.5 (SD)		
Rainfall (mm)	0.0686	-
Minimum Temperature (°C)	−0.0071	0.0704
Vapor Pressure (hPa)	0.0426	−0.0184

We find that rainfall has a positive relationship with diarrhea in the wet season and no relationship in the dry season. Minimum temperature and vapor pressure have a bidirectional influence on diarrheal case incidence by season. Vapor pressure positively affects wet season diarrheal case incidence but negatively impacts dry season diarrheal case incidence, with minimum temperature having the opposite seasonal effects. 

## 4. Discussion

**Figure 9:**
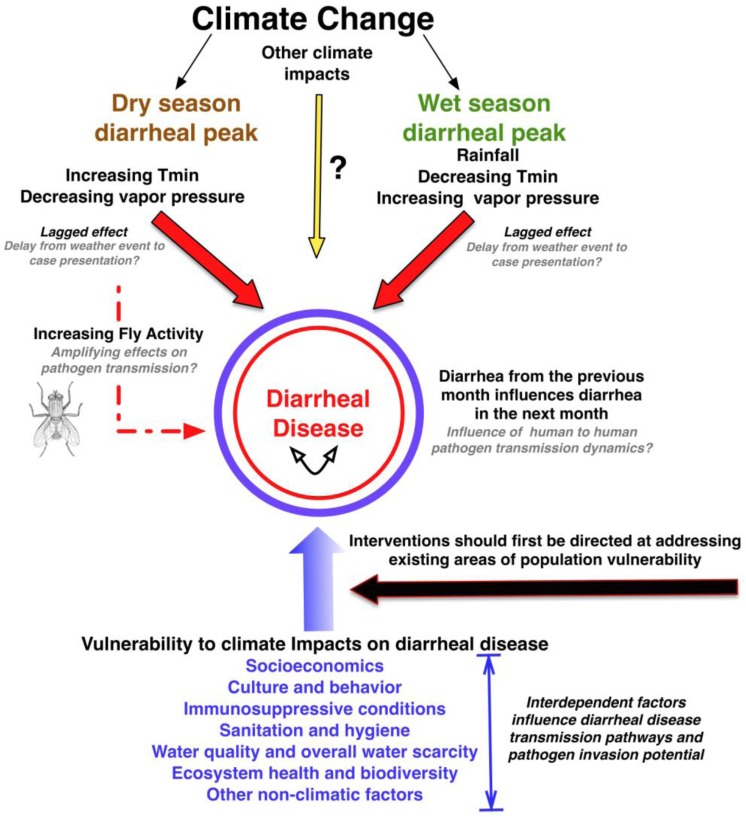
Schematic illustration of the potential interactions between climate change, seasonal diarrheal peaks, meteorological variables, other non-climatic factors, and diarrheal disease in Botswana. Favorable climatic conditions in the dry season (hot and dry) can influence fly population density and activity, possibly contributing to enhanced pathogen transmission during this period. Interventions that minimize factors contributing to population vulnerabilities will contribute to present day public health needs and reduce population sensitivity to future climate change impacts on this disease syndrome.

Our study identifies significant climate-diarrhea interactions likely to be negatively influenced by forecasted climate changes for the region. Diarrheal case incidence peaks biannually in the wet and dry seasons in Botswana with mean case incidence highest in the dry season. Diarrheal case incidence during the study period was significantly correlated with diarrhea cases in the previous month and key one month lagged climatic variables: T_min_, vapor pressure (q), rainfall, and the derived variable, T_min_/q (Figure 6, Figure 9).

In our model, T_min_, vapor pressure, and rainfall accurately predict up to 58% of the variability of future diarrhea case incidence (Figure 8) identifying the importance of climatic influences on diarrheal disease in Botswana. We now discuss identified climate-diarrheal interactions and potential climate change impacts, study limitations and caveats, as well as provide recommendations for future research directions and health management.

### 4.1. One Month Lag in Relationships between Model Variables and Diarrhea Case Incidence

Modeled interactions between meteorological variables and diarrheal disease case incidence were strongest with a one-month lag in relationships. The data were aggregated at the month level so finer temporal associations could not be identified in our assessment. Time delays between weather events and diarrheal disease have been reported in other studies examining the relationship between meteorological variables and diarrheal disease incidence [30,31,32,33]. Lagged relationships may represent time delays between weather events, environmental change, pathogen exposure, invasion, and incubation periods, and the onset of clinical signs of diarrheal disease. 

There was also a strong positive autocorrelation in diarrheal case incidences at the one-month lag level. In other words, diarrheal case incidence in the previous month influenced case incidence in the following month. Pathogen transmission is the primary factor characterizing infectious disease dynamics. For many pathogens, the number of infected hosts will influence the rate at which susceptible hosts are exposed to infectious material and, consequently, become infected. This process may contribute to the dependency observed between previous and current month’s diarrheal case incidence. This suggests that in addition to other drivers of diarrheal disease, pathogen shedding by infected individuals in the system may contribute importantly to subsequent diarrheal cases and that interventions directed at breaking these transmission pathways may contribute to a reduction in diarrheal incidence (e.g., removal of excreta, strengthening of sanitation). 

### 4.2. Dry Season Diarrhea

In other studies, diarrheal disease was increased in the wet season, a phenomena speculated to be associated with contamination of surface water (reviewed in [34]). In contrast, diarrheal incidence in Botswana is increased in the dry season by an average of 20% over wet season diarrhea. This may be a function of the arid nature of Botswana where there is limited surface water available in the country (Figure 1). 

While increases in temperature have been previously identified as an important consequence of climate change, minimum temperatures have been identified as a more sensitive indicator having increased twice as fast as maximum temperatures in many regions [35,36]. In our study, minimum temperature was the only temperature parameter positively associated with diarrheal disease over the study period. This may be related to the subtropical climate of Botswana where temperatures, irrespective of season, generally rise during the day (average maximum temperature range 22.17–35.72 °C). Minimum temperatures, on the other hand, vary more significantly by season from 3.16 °C in the cold dry season to 21.39 °C in the hot dry season. Vapor pressure was also associated with diarrheal disease in the dry season with lower vapor pressure positively associated with increased diarrheal disease. 

When we consider both of these variables together (assessed as minimum temperature divided by vapor pressure), the highest value for this derived variable is in October, identifying this month as the hottest and driest month of the year in the country. This is also the peak for dry season diarrheal disease at the national level (Figure 6 (a)). The reasons for this are uncertain, but may be influenced by changes in water quality, hygiene related to water economizing, sanitation, increased water intake and sharing of water utensils, food spoilage, and increases in fly activity and density, which is discussed separately below. 

Our results are very different than the dry season findings of Bandyopadhyay and colleagues [10] in their study of diarrheal disease and climate in 14 countries in sub-Saharan Africa. In this study, increases in maximum temperature were associated with increased prevalence of diarrhea, a variable that was not significantly associated with diarrheal disease in our study. More importantly, their study identified increases in minimum temperature as being associated with reduced diarrheal illness. The authors speculate about the potential influence of rotavirus transmission dynamics on this observation. In our study, we found the opposite, with elevations in T_min_ to be positively associated with increased diarrhea disease incidence in the dry hot season, but negatively associated with diarrheal disease in the wet season. Another 12-year study in neighboring Limpopo Province in South Africa found that maximum temperature and rainfall were significantly associated with childhood diseases including diarrheal disease [37]. These contrasting results suggest that spatially focused, long-term time series data are likely necessary to clearly identify climate-diarrhea relationships in Africa. 

#### Climate Influences on Fly Abundance and Dry Season Diarrheal Disease

Of interest is the potential relationship between the climate, diarrheal disease, and fly abundance and activity. There has been significant attention directed to the potential influence of climate change on the distribution of vectors such as ticks and mosquitos and increases in associated vector borne disease [38,39]. However, few studies have been directed at evaluating climate change impacts on fly abundance and the mechanical transmission of infectious disease despite the importance of flies as mechanical vectors of diarrheal disease-causing microorganisms [40,41,42]. 

Calyptrate fly species (Family: Sarcophagidae (flesh flies), Muscidae (house flies and latrine flies), and Calliphoridae (blowflies and bottleflies)) are adapted to living in proximity to humans (i.e., synanthropic) and are important mechanical vectors of pathogenic bacterial, fungal, protozoal, and viral pathogens, many of which can induce diarrheal disease [43,44,45,46,47]. *Musca domestica*, the common housefly is found widely in Botswana [48] along with other calyptrate fly species. This species is able to respond rapidly to environmental change with adult population size, under appropriate conditions, able to double in only a few days [49,50]. Calyptrate species have been shown to be maximally active when temperatures are high and humidity and rainfall are low [51,52]. Indeed, a recent study identifies that increases in temperatures associated with forecasted climate change will dramatically increase fly population density with *M. domestica*, for example, increasing 244% by 2080 under the worst-case climatic scenario, and by 156% under the moderately optimistic medium-low emissions scenario [52]. 

Dry season diarrheal disease peaks in Botswana may then, in part, be related to the direct influence of high T_min_/q and on both fly activity and density with enhanced environmental dissemination of diarrhea-inducing microorganisms and increased human exposure. Flies in this system may provide a dry season amplifying influence on existing sanitation and hygiene deficiencies and other factors contributing to diarrheal disease in this country (Figure 9). There is an urgent need to refine our understanding of the potential interaction between climate, flies, and diarrheal disease and forecasted climate change impacts in Africa. 

### 4.3. Wet Season Diarrhea

Previous studies have found a positive relationship between rainfall and diarrheal disease [7,8,10,11]. In our study, lagged rainfall and vapor pressure were both important predictors of diarrheal disease. Many studies reporting correlations between diarrheal disease and rainfall do not include an evaluation of vapor pressure [8,10,53], perhaps an important but neglected climate variable in many studies attempting to model diarrheal-climate interactions. While rainfall and vapor pressure are related environmental indices, they measure importantly different information on both spatial and temporal scales, particularly in subtropical regions. Water vapor pressure is defined as the atmospheric pressure exerted by water vapor (water in its gaseous state) [54]. It is one way of measuring the humidity of the air. At a given temperature, an increase of water vapor in the air corresponds to an increase in the humidity of the air. In meteorology, precipitation (rainfall being a form of precipitation) is any product of the condensation of atmospheric water vapor that falls under gravity [54]. Botswana lies almost entirely within the tropical wet-dry bioclimatic type [55], which is characterized by marked seasonal contrasts between the wet and dry season, driven mainly by seasonal migrations of low and high-pressure features. During the dry season, subtropical high pressure associated with regional subsidence dominates, suppressing the precipitation formation process. In contrast, the wet season is associated with a regional presence of the Intertropical Convergence Zone (ITCZ) and its associated low pressure [56]. This low-pressure trough is associated with a rapid convective lift, resulting in a precipitation climatology characterized by short duration but intense events that are often quite localized. Vapor pressure, on the other hand, measures moisture content in the lower atmosphere, which is continually being mixed by horizontal and vertical motion. It is, therefore, a more spatially and temporally homogeneous, “smoothed” metric that might not reflect variable finer scale rainfall events that may or may not be captured if the observation network is sparse or non-random. Evaluation of rainfall and vapor pressure provide different and important insights into climate-health interactions.

Both rainfall and vapor pressure can have impacts on pathogen transmission and survival, and diarrheal disease incidence. Rainfall events can influence the movement of pathogens from environmental reservoirs into ground water, surface water, or drive contamination of water systems. For example, unusually high rainfall and floods were associated previously in Botswana with suspected sewage contamination of the environment and major diarrheal disease in the eastern part of the country. More than 547 children under the age of five years died in the eastern part of the country during a 2006 outbreak of diarrheal disease associated with unusually heavy rains [57]. Vapor pressure on the other hand may influence survival of diarrhea causing microorganisms in the environment although effects tend to be species specific with both negative and positive survival impacts [58,59]. While rainfall is only significant in the wet season, vapor pressure’s bidirectional influence on diarrheal case incidence in both the wet and dry seasons identify complexity in this climate variable and the need for more in-depth studies identifying the mechanism of influence on diarrheal disease occurrence. 

### 4.4. Long Term Trends in Diarrheal Disease

Over the 30-year study period, annual diarrheal case incidence averaged 88.47 ± 16 cases per year per 1,000 people. Reported diarrheal case incidence has decreased since 1974 after accounting for increases in the availability of health facilities. This decline in incidence over time is likely related in part to the infrastructural investment and development of sanitation and water delivery infrastructure health services, and education, which has been dramatic in Botswana since acquiring independence.

### 4.5. Climate Change and Diarrheal Disease in Botswana

Climate change in Botswana is already evident with an average increase in annual daily temperatures of 0.089 °C per annum between 1981 and 2011 [60]. Under climate change influences, temperature is projected to increase from 2.5–3 °C [61] and the country to become drier by 5–15% [62,63]. Pacific El Nino Southern Oscillation (ENSO) episodes are forecasted to increase in frequency and associated droughts will be more intense [64]. With a 10% reduction in rainfall, models suggest that perennial drainage will be significantly reduced, affecting already limited surface water resources [65]. Our analysis of historical data suggests that these climate change projections of a drier, hotter climate will possibly exhibit a positive influence on dry season diarrheal case incidence in Botswana (Figure 9). The importance of this potential impact is seen in our historical data with the highest year of diarrheal case incidence across the data set occurring in 1992, a drought year noted for being one of the harshest for Southern Africa [66,67]. In contrast, forecasted declines in rainfall are likely to have a negative effect on diarrheal incidence in the wet season. Although uncertain, declining rainfall patterns may enhance dry season diarrheal incidence with dry, hot conditions starting earlier and lasting longer into the wet season period (Figure 9). 

### 4.6. Climate Change and Interacting Impacts

Climate change health impacts are not likely to arise in isolation. Identified climate-disease linkages may be amplified by other forecasted climate change impacts. Climate change forecasts of increased drought and decreased rainfall associated with climate change are predicted to drastically reduce agricultural production in Botswana along with surrounding countries, affecting regional nutrition and food security [68]. Malnutrition can substantially influence both disease susceptibility [69] and the risk of diarrheal disease in children under five [70,71], potentially increasing the impact of climate change on this disease syndrome. 

### 4.7. Water, Arid Countries, and Diarrheal Disease

The quality and quantity of readily available water is particularly important in diarrheal disease and can identify direct exposure to water-borne pathogens as well as significantly influence hygiene and sanitation practices [72]. Water availability and quality are increasingly being affected in arid countries with shortages and limited distribution systems a continuing challenge, particularly in Botswana [73], Alexander unpublished data]. Indeed, over 1.1 billion people across the globe do not have access to safe drinking water. The greatest limitations in water access are identified in sub-Saharan Africa, where 44% of the people do not have access to safe water sources [74]. Even when clean water is available, escalating poverty means many still cannot afford the connection fees associated with these often privatized services [75,76]. Climate change is predicted to further reduce water availability, impacting access in Southern Africa [62]. While climate change is considered an important and increasing threat, current human impacts on limited fresh water resources are identified as an existing critical problem [77] particularly in Botswana [78]. Addressing current water deficiencies in Botswana and other arid countries through enhanced regional water planning (access and quality) and water conservation practices will be an essential first step towards mitigation of present health challenges and potential future climate change impacts. Diarrheal-climate interactions identified in this study highlight the need for urgent strategic action in this area.

### 4.8. Study Limitations and Caveats

#### 4.8.1. Data Acquired from Passive Surveillance Systems

Data obtained from passive surveillance systems may have weaknesses that obscure important spatial and temporal characteristics of an infectious disease as well as the magnitude and severity of an outbreak. Acute infectious diarrhea is generally considered to be underreported [79]. Clinical signs of diarrhea from a particular causative agent may vary from mild to severe (e.g., *Campylobacter*) or infections may present asymptomatically (e.g., giardia, see [79]) decreasing health facility visitation and disease reports, although incidence of infection may not have changed. The number of health facilities may also strongly influence diarrheal case reporting over time as is the case in our study, where the number of diarrheal case reports was positively associated with increases in health facility development (Figure 4).

Cultural sensitivities on the discussion and reporting of diarrhea within a family may also influence within household health-seeking behavior. For example, older children may not report diarrheal disease to caregivers until the disease is noticeably severe (Alexander unpublished data). Without such communication, a visit to a health facility will not occur. Cultural preference for traditional healers or remedies may influence health seeking behavior according to age and/or gender at the level of the individual or the entire household [80,81]. In Botswana, the use of traditional or western health approaches was further influenced by culturally based views and understanding of disease causation or source [82]. Health-seeking behavior in general can be influenced by a variety of broad reaching factors related to social infrastructure, education, demographics, religious belief systems, status of women and children, economic and political systems, environmental conditions, distance to health facilities, nature of the disease, and institutional approaches identified in the public health care system (for a review see [83] and also [84,85,86]). These factors can vary significantly by household and region, potentially reducing surveillance sensitivity in certain areas, skewing assessments of disease incidence. 

While not a factor in this study, political stability and economic declines can have serious impacts on public health infrastructure, health care, and the accuracy of disease surveillance systems. These impacts can influence disease outbreaks directly (e.g., cholera in Zimbabwe [87]) as well as compromise surveillance and reporting activities, undermining the validity of these longitudinal data sets that include periods of weakened governance. Economic development and infrastructure, training, work practices, and motivation of medical staff can also greatly influence the quality of data collected, the impacts of which are difficult to identify in passive data collection systems. While some of these limitations can be minimized (see Experimental Section, data transformation), ultimately, the performance of a particular passive surveillance system in terms of data accuracy (sensitivity and specificity) and precision (repeatability) must be considered. Data, however, derived from passive surveillance systems, particularity in Africa, represent often the only source of systematically collected health information. 

#### 4.8.2. Complexity of Diarrheal Disease Causation and Transmission Dynamics

Diarrhea as a syndrome can also be caused by a wide variety of pathogens with seasonality and climate interactions differing by etiological agent [88,89]. Patterns of occurrence are further influenced by pathogen transmission dynamics shaped by seasonal factors, pathogen and host community characteristics and involvement of zoonotic pathogen sources, and environmental conditions. Transmission of diarrhea causing pathogens from infected to susceptible hosts can occur over a multitude of interdependent pathways [90], either through direct transmission between hosts, and/or indirectly through vectors, water, food, fomites, and/or other sources of environmental contamination [91,92,93,94]. These chains of transmission can involve human-human, animal-human (zoonotic), and/or human-animal-human (zoonotic and anthropozoonotic) transmission linkages and environmental transmission pathways. Within environments where multiple pathogens may contribute to diarrheal diseases, these complexities can mask epidemic signals emanating from a single pathogen or complicate and obscure finer pathogen-meteorological interactions. Notwithstanding the complexities in causation and transmission dynamics, this study identifies a strong climatic signature in diarrheal disease case incidence in Botswana. 

#### 4.8.3. Socioeconomic Impacts

Diarrheal disease can be strongly influenced by socioeconomic factors including sanitation and hygiene practices and use of unsafe water sources [11,72,95,96]. Nutritional status among children (the most affected group), for example, can also influence disease susceptibility and diarrheal disease [97]. In Botswana, this can be considered largely a constant across the country and sampling period, as the Botswana Government, since the early 1980s, has monitored and provided free supplementary foods to any child with malnutrition [98] or those nutritionally vulnerable such as orphans associated with the human immunodeficiency virus/acquired immunodeficiency syndrome (HIV/AIDS) epidemic [99]. However, these socioeconomic influences must be considered when evaluating climate-diarrhea interactions using longitudinal data sets. In this study, we standardized monthly data by year to minimize the influence of these factors over time (see Section 2). Ultimately, however, the myriad of factors that can influence health facility disease reporting highlight the potential limitations to the evaluation of long-term trends. Our study results illustrate the importance of considering potential sources of bias and confounders in longitudinal data sets and approaches that might be applied to minimize the influence of these factors.

#### 4.8.4. HIV/AIDS

The spatial occurrence of diarrheal disease can also be influenced strongly by the concurrent presence of other infectious diseases. Botswana has one of the highest HIV prevalence levels in the world (36% [100]). This epidemic has impacted population vulnerability to infectious disease generally and diarrheal disease in particular [101]. Paradoxically, some health interventions designed to decrease the transmission of HIV/AIDS appear to contribute to the occurrence of diarrheal illness [57,102]. For example, natural maternal immunity, important in fighting water-associated infections, is absent in a great percentage of children in Africa, particularly in Botswana as HIV-positive mothers are advised to use formula rather than breast-feed to reduce mother-to-child transmission of the virus [103]. In 2006, in the wet season in Botswana, over 35,000 diarrhea cases and 532 deaths were reported among children less than 5 years of age in association with heavy rainfall and flooding. Affected children in this outbreak were less likely to have been breast fed [102]. This interaction highlights the complexity of diarrheal disease causation and the difficulty in predicting the scope of climate change health impacts. 

#### 4.8.5. Scale of Study

While future climate scenarios can be applied to national data sets to estimate the burden of disease associated with climate change [104], it is important to note that national evaluations may mask finer scale spatial variation important in predicting local risk and vulnerability. In particular, fine-scale interactions between hydrology and land use may have important influences on local climate and future climate change vulnerabilities [12]. For example, most of Botswana is without permanent surface water (Figure 1) and areas with surface water (e.g., Chobe District in northern Botswana) have a different dry season pattern of diarrheal disease than at the national level (Alexander, unpublished data). The reasons for this variation are unknown but appear related in part to hydrology of the river system and resultant changes in water quality. 

The complexity of interactions (socioeconomic and environmental) and impacts on diarrheal disease mean that studies such as this provide important large-scale qualitative predictions on the influence of climate on diarrheal disease. These models, however, are unlikely to provide accurate quantitative predictions regarding changes in diarrhea in response to climate. Rather, study outputs provide a directional assessment of impacts important in the development of national research and public health agendas as well as supporting construction of refined hypotheses regarding climate-health interactions.

### 4.9. Predictions of Climate Change Impacts on Health—The Importance of Non-Climatic Factors

A study conducted by Gething and colleagues [105] provides an important cautionary message regarding the importance of considering the full context of disease dynamics in predicting the effect of climate change impacts. Malarial disease is predicted to increase with increasing temperature. Their assessment over the last century identified a global decrease in spatial extent and incidence of malaria despite increases in temperature occurring during that period. Human development and disease control responses are thought to have influenced this contraction. The authors and others [106,107] emphasize the important influence of other non-climatic factors on biological phenomena and the potential for these interactions to have a greater impact on disease dynamics than that predicted by climate change. 

Thus, while our work identifies important climate-health interactions and increased vulnerability of Botswana to forecasted changes in regional climate, diarrheal trends may not be influenced as predicted. Our findings do not account for the effect of non-climatic factors such as improved sanitation and hygiene on pathogen transmission pathways and the potential for this to dampen climate-diarrheal disease interactions. The impact of forecasted climate change on this disease syndrome is likely to be significantly reduced if present day public health deficiencies are fully identified and effectively addressed. Understanding how climate interacts with disease transmission and persistence dynamics will be critical to intervention design and underscores the importance of climate-health research in addressing present-day public health needs and preparing for future climate change impacts.

### 4.10. Public Health Interventions, Population Vulnerability, and Diarrheal Disease

Diarrheal disease management approaches must consider the interdependency of transmission pathways and the complexity of interacting drivers related to culture and behavior, socioeconomics, and system ecology [90,108,109]. These drivers also influence the nature of human population dependencies and couplings to the natural environment and resultant feedback processes. These interactions can strongly influence the sensitivity of a population to climatic processes and environmental change. Interventions directed at addressing these underlying vulnerabilities will provide importantly to improved public health and climate change preparedness (Figure 9). Intervention adoption by locally communities, however, will depend on their perception of risk, benefits, and cultural appropriateness of the intervention approaches, underscoring the need to include communities in public health strategy development and intervention design.

## 5. Conclusions

Climatic drivers (T_min_, vapor pressure (q), and rainfall) influence diarrheal disease occurrence and highlight the potential vulnerability of Botswana to increased diarrheal disease incidence under forecasted climate change scenarios (Figure 9). Study findings have application to other arid countries in Africa where diarrheal disease is a persistent public health problem. Identifying in-country spatial heterogeneities and risk-enhancing environmental factors that will influence climate-diarrheal disease interactions must be a priority. There is also an urgent need to better understand climate-fly-diarrhea dynamics particularly as potential interactions are likely to be impacted by forecasted increases in temperature. Identifying potentially synergistic influences of other projected climate impacts on health outcomes will be an important challenge to the full identification of health vulnerabilities across the country. 

In arid regions such as Botswana, water resource restrictions and their impacts on health, sanitation, and hygiene identify an increasingly significant problem. With water quality, access, and sanitation challenges already compromising community health in many locations in the country, our findings indicate that climate change is expected to only enhance these current health challenges. Current deficiencies must be addressed with urgency. 

Finally, public health strategies directed at reducing population vulnerability to diarrheal disease and future climate change impacts must be locally acceptable and culturally appropriate. Lack of inclusion of sociocultural considerations into public health planning will most likely result in locally applied interventions being non-sustainable. 

Our work identifies urgency in refining our understanding of both climatic and non-climatic drivers of diarrheal disease in order to manage present day health challenges and potential future climate change impacts. 
